# Review of the Leaf Essential Oils of the Genus *Backhousia* Sens. Lat. and a Report on the Leaf Essential Oils of *B. gundarara* and *B. tetraptera*

**DOI:** 10.3390/plants11091231

**Published:** 2022-05-02

**Authors:** Bruce F. Bowden, Joseph J. Brophy, Betsy R. Jackes

**Affiliations:** 1 College of Science and Engineering, James Cook University, Townsville, QLD 4811, Australia; bruce.bowden@jcu.edu.au (B.F.B.); betsy.jackes@jcu.edu.au (B.R.J.); 2 School of Chemistry, University of New South Wales, Sydney, NSW 2052, Australia

**Keywords:** *Backhousia*, essential oils, *Choricarpia*, *Syzygium anisatum*

## Abstract

A review of the leaf oils of the 13 species now recognised in the genus *Backhousia* is presented. This review carries on from, and incorporates data from, an earlier (1995) review of the then recognised eight species. The leaf oils of two new species of *Backhousia*, *B. gundarara* and *B. tetraptera* are reported for the first time. *B. gundarara* contains a mixture of mono- and sesquiterpenes, with α-pinene (14%) and spathulenol (11%) being the main members. In *B. tetraptera*, the principal component of the mainly terpenoid leaf oil is myrtenyl acetate (20–40%). The review also incorporates the two species of the genus *Choricarpia*, which have been subsumed into *Backhousia*, *viz. B. leptopetala* and *B. subargentea*. Due to its history in *Backhousia*, *Syzygium anisatum*, which has been transferred out of *Backhousia*, is included in the review for historical reasons.

## 1. Introduction

*Backhousia* Hook. & Harv. is a genus currently comprised of 13 species, within the Family Myrtaceae, sub family Myrtoideae [1]. It is now the sole member of Tribe Backhousieae. It was first described in Curtis’ Botanical Magazine in 1845 by William Jackson Hooker and William Henry Harvey. The species so named was *Backhousia myrtifolia* Hook. & Harv. As the authors report:

“This very pretty greenhouse shrub, its conspicuous petalois calycine segments giving the idea at first sight of large corollas to flowers, was found by Mr. James Backhouse in the Illawara (*sic*) district of New South Wales; and, not being referable to any Myrtaceous Genus yet described, Mr. Harvey and myself are anxious to dedicate it to our mutual friend now mentioned, who, amidst his various and arduous labors of love during a voyage to, and journeyings in, various parts of Australia and South Africa, still found leisure to collect and to describe in manuscript many interesting plants, which his previous botanical acquirements enabled him to do with great judgement” [2].

The species in this genus grow as aromatic shrubs or trees (5–25 m tall), with leaves 3–12 cm long and 1–6 cm wide, which are arranged opposite to each other. The genus is represented, with one exception, as endemic to the rain forests and forests of eastern Australia (New South Wales and Queensland). Recently, one species has been identified from the Kimberley region of Western Australia.

The first report of leaf oils from the genus was in 1888, by the firm Schimmel & Co of Miltitz, Germany, who reported that the leaf oil of *B. citriodora* was almost exclusively (95%) citral [3].

The leaf oil of *B. myrtifolia*, from which the genus was named, was investigated by Penfold in 1922 [4]. Later work by Penfold et al. in 1953 [5] showed the existence of physiological forms. Still, later work by Hellyer et al. in 1955 looked at individual trees [6] and confirmed the occurrence of four forms based on the oils containing major amounts of the aromatic ethers methyl eugenol, methyl isoeugenol, elemicin and isoelemicin.

Jones and Lahey, in 1938, examined the leaf oil of *B. hughesii* [7], and *B. bancroftii* in 1939 [8], identifying both D-α-pinene and D-β-pinene in the former species and only α-pinene in the latter because of the poor oil yield of oil in the latter species.

Penfold, in 1923, first examined the leaf oil of *B. angustifolia* and found ‘phenolic’ compounds [9]. This led to a significant amount of chemistry by Birch in 1951 [10], and colleagues in 1954 [11], 1956 [12], Cannon and Corbett in 1962 [13] and Brophy et al. in 1989 [14]. All examined the phenolics, β-triketones and ketones, while looking at the physiological forms of this species.

In 1949, McKern reported on the presence of anethole in the leaf oil of what was then called *Backhousia anisata* [15]. Later work by Brophy and Boland in 1991 revealed the existence of two physiological forms, an anethole form and a second less frequent methyl chavicol form [16]. Later work by Southwell et al. in 1996 [17] and Blewitt and Southwell in 2000 [18] further examined these physiological forms and their occurrence in the field. The species was renamed *Syzygium anisatum* in 2005 [19], after being originally moved to a monotypic genus *Anetholea* [20].

Some 30 years after the initial report in 1888 that *B. citriodora* oil contained 95% citral [3], Blogg used the oil as a source of citral for conversion to the more valuable ionone [21]. A further 30 years later, Penfold et al. discovered the existence of physiological forms of *B. citriodora* [22,23]. In the second physiological form, L-citronellal accounted for up to 75% of the oil. This was a much rarer form. It took almost 50 more years for this L-citronellal form to be re-discovered [24,25], and it appeared to be arising from a single parent tree. The story of the oil of this species has been well-serviced by a recent review by Southwell [26].

Penfold reported on the leaf oil of *B. sciadophora* in 1924 [27]. Bean, in 2003, named *B. oligantha* [28], a species whose oil was examined by Brophy et al. in 1995, as *Backhousia* sp. (Didcot P.I. Forster PIF12671) [29].

The leaf oil of *B. kingii*, which had been separated from *B. sciadophora* in 1988 by Guymer [30], produced an oil very similar to the species from which it was split [29].

Recently, two new species of *Backhousia*, *B. gundarara* and *B. tetraptera* have been reported [1]. Their leaf oils are reported on for the first time in this publication. One of these species, *B. gundarara*, occurs in two small isolated locations at the base of cliffs in the isolated Kimberley region of Western Australia.

In the same publication that *B. gundarara* and *B. tetraptera* were named, the two species of *Choricarpia* were subsumed into the genus *Backhousia*, as *B. subargentea* and *B. leptopetala* on the basis of DNA analyses [1]. The leaf oils of these two species, whose major components are quite similar, have been previously reported on, as *Choricarpia*, in 1994 [31].

What follows is a report of the contents of the leaf oils of the known species of *Backhousia*, whose leaf oils have been published over the last 100 years, together with a description of the leaf oils of the newly named species, *B. gundarara* and *B. tetraptera,* and the recently transferred species, *B. subargentea* and *B. leptopetala.* The leaf oils of *Syzygium anisatum* are also included in this review because of their history in the genus *Backhousia*. This review incorporates data from a previous publication (1995) by Brophy et al. on the leaf oils of the genus *Backhousia* [29].

In all cases, the components are reported in the order that they elute from a polar glc column.

## 2. Results

### 2.1. Backhousia gundarara M.D. Barrett, Craven & R.I. Barrett

**Figure 1:**
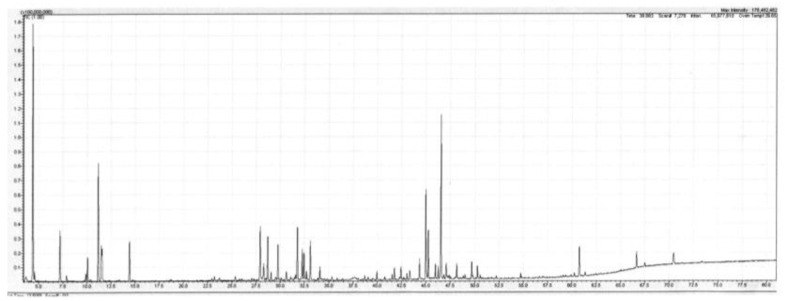
Total ion current (TIC) trace of the leaf oil from *Backhousia gundarara* on a polar column. Conditions are given in the Experimental section.

*Backhousia gundarara* M.D. Barett, Craven & R.I. Barrett is a shrub or small tree with smooth bark that grows up to 7 m in height. It was discovered by W. O’Sullivan and D. Dereau in the upper reaches of the Prince Regent River, Western Australia in 2001. A second collection was made by G. and N. Sankowsky, also in the upper region of the Prince Regent River in 2003. It is known only from these two locations, in the Kimberley region of Western Australia. The leaf oil sample described below originates from the Sankowsky collection. This species is the only species of *Backhousia* not to occur naturally on the east coast of Australia, although it is growing at Tolga in north Queensland from cuttings taken during the Sankowsky collection. The leaf oil of *B. gundarara*, produced in 0.3% yield (*w*/*w* fresh leaf), contained a mixture of mono- and sesquiterpenes in approximately equal amounts. Additionally, present were six aromatic compounds (from their mass spectra) totalling approx. 9% of the oil. These remain unidentified at the moment. The main monoterpenes were the hydrocarbons α-pinene (13.6%), limonene (3.6%), and p-cymene (1.2%). The oxygenated monoterpenes were not as plentiful, with the principal members being terpinene-4-ol (1.5%) and α-terpineol (1.2%). Of the sesquiterpenes, the major compounds were the alcohols globulol (6.1%), viridiflorol (3.3%) and spathulenol (11.1%). Of the hydrocarbons the main compounds were aromadendrene (1.6%), viridiflorene (1.2%) and an unknown sesquiterpene hydrocarbon (unknown X), whose mass spectrum is given in the footnotes to Table 1 (2.1%). Additionally, present in the oil was what is suspected, from mass spectrum and linear retention index (LRI) data, of being 2,4,6-trimethoxytoluene (0.4%). A detailed list of compounds identified in the oil is set out in Table 1 below, and a Total Ion Current (TIC) trace of the leaf oil from *B. gundarara* on a polar column is given in Figure 1.

### 2.2. Backhousia tetraptera Jackes

*Backhousia tetraptera* Jackes is a newly described species growing in gullies on Mount Stuart, Townsville, Qld, at an altitude of about 500 m. It occurs as a population of 170–180 trees. A second site has recently been found at Clement State Forest, near Rollingstone, Qld. It is a tree, usually growing 5–8 m in height, but can grow up to 15 m. It has leaves 5.5–9 cm in length and 1.5–3.8 cm in width. Oil glands are rather sparse in the leaves, and it was not surprising that the oil yield on steam distillation was low, 0.1–0.2%, *w*/*w* fresh weight leaves.

Three collections of *B. tetraptera* foliage were available for steam distillation: one individual tree, a bulk of 3 trees and a sample grown from seed from an individual tree. The oils obtained from the three samples were similar. There were a considerable number of monoterpenes present in this oil, though sesquiterpenes were also well represented. The main component was the ester myrtenyl acetate (24–46%). This ester was accompanied by lesser amounts of α-pinene (3.7–3.8%), linalool (5.0–8.9%), myrtenol (0.5–2.1%) and α-terpineol (0.3–0.7%).

**Figure 2:**
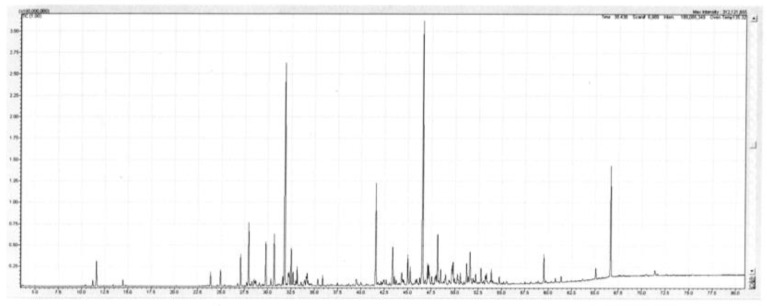
TIC trace on a polar column of the leaf oil of *Backhousia tetraptera.* Conditions are given in the Experimental section.

The main sesquiterpenes encountered were the hydrocarbons β-caryophyllene (1.4–5.0%), allo-aromadendrene (0.7–1.5%), α-humulene (0.8–2.3%), viridiflorene (1.9–6.3%), and α-selinene (0.7–2.3%). The main oxygenated sesquiterpenes were caryophyllene oxide (2.6–3.7%), humulene oxide II (1.2–1.6%), globulol (0.8–1.4%), and spathulenol (7.0–8.5%). There were also small amounts of the aromatic ethers methyl eugenol (tr-0.2%), eugenol (0.9–1.2%) and 2,4,6-trimethoxytoluene (0.6–0.9%). A detailed list of the compounds identified in the oil is set out below in Table 2. A TIC trace on a polar column of the leaf oil of *Backhousia tetraptera* is given in Figure 2.

### 2.3. Backhousia angustifolia F.Muell.

*Backhousia angustifolia* F.Muell. is a shrub or small tree growing up to 7 m height, which occurs in semi-evergreen vine thickets in the Burnett, Darling Down, Wide Bay, Leichhardt, North and South Kennedy, Cook, Moreton and Port Curtis pastoral districts of central and southern Queensland [33]. The species was first investigated by Penfold et al. [9] in 1923, who found that the essential oil contained 75% of phenolic material while the remaining 25% comprised monoterpenes (D-α-pinene, β-pinene, 1,8-cineole, alcohols including α-terpineol and sesquiterpenes) and a stearoptene, which was later shown by Birch et al. [10] to be angustifolionol (5-hydroxy-7-methoxy-2,6,8-trimethylbenzochromone) (1). The phenolic material was later shown to be a mixture of two β-triketones, angustione (2) and dehydroangustione (3) [11,12]. Cannon and Corbett [13] showed that *B. angustifolia* consisted of at least two physiological varieties depending on whether angustifolionol (1) was present or not. More recently, Brophy et al. [14] identified a new ketone, angustifolenone (4) (D-2-ethyl-4,6,6-trimethylcyclohex-2-ene-1-one) in the most northly occurrence of this species. The species has been shown to have antibacterial properties [33]. Triterpenes of *B. angustifolia* have also been investigated [34]. Brophy et al. also found that the β-triketone platyphyllol (2-acetyl-4,6,6-trimethyl-5-methoxycyclohex-4-en-1,3-dione), first identified in the oils of *Melaleuca cajuputi* subsp. *platyphylla* [35], occurred in small amounts in most collections. The mass spectra of many of these compounds have also been published [36].

Five chemical varieties have tentatively been suggested in this species. These were:

- Oils containing angustifolionol (5-hydroxy-7-methoxy-2,6,8-trimethylbenzochromone) (1) in large amounts;
- Oils containing a large amount of angustione (2);
- Oils containing a large amount of dehydroangustione (3);
- Oils containing significant amounts of angustifolenone (2-ethyl-4,6,6-trimethylcyclohex-2-en-1-one) (4) and dehydroangustione (3) with only small amounts of angustione (2);
- A form from southern Queensland whose oil contained significant amounts of E-β-ocimene, 1,8-cineole and angustione (2).

**Figure 3:**
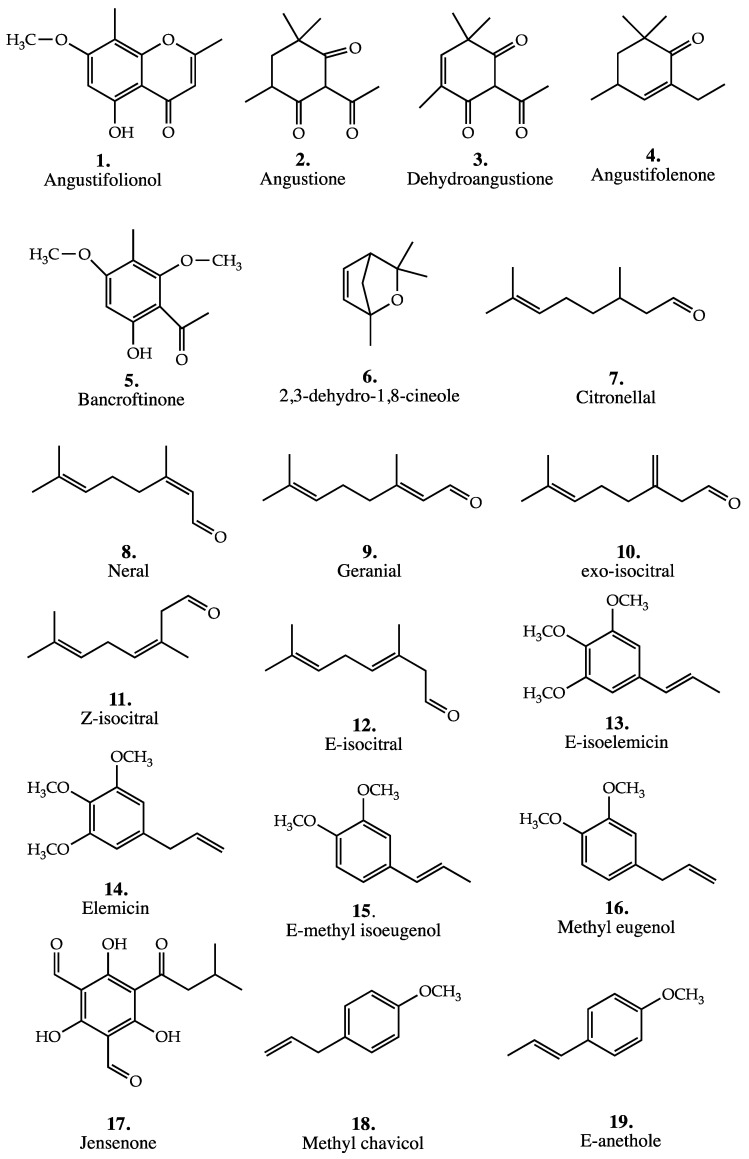
Structures of major or diagnostic compounds mentioned in this review for all *Backhousia* species.

The structures of these compounds are shown in Figure 3. The composition of the different varieties are given in Table 3 below.

### 2.4. Backhousia bancroftii F.M.Bailey

*Backhousia bancroftii* F.M.Bailey is a rainforest tree growing up to 25 m in height [37]. It occurs in the Cook and north Kennedy pastoral districts of tropical Queensland. The oil yield from this species was poor (0.03–0.10%) based on fresh leaves. Before the analyses by Brophy et al. [29] the only report on the leaf oil was in 1939, where Lahey and Jones found very poor oil yields and sesquiterpenes as the principal components, with α-pinene and esters as minor constituents [8].

Brophy et al. [29] found that the principal components of the oils of this species were terpenes (mainly sesquiterpenes), alkyl derivatives (alcohols and esters, mainly acetates) and aromatic compounds. There was significant between-tree variation in the oils.

In all but one of the trees examined, the main components were alkyl acetates: in the majority of trees, it was octyl acetate (33–62%), but in one bulk sample it was decyl acetate, with another tree containing approximately equal amounts of decyl- and dodecyl acetates and the corresponding alcohols. In all the oil samples, octyl-, decyl-, dodecyl- and tetradecyl acetates and the corresponding alcohols were significant components, between them accounting for the majority of the leaf oil. There were also small amounts of higher esters identified by mass spectrometry.

Terpenes were very minor components, two trees containing α-pinene, but all trees contained small amounts of sesquiterpenes (both hydrocarbons and oxygenated). In all cases they were individually <3%.

The two principal aromatic compounds identified in the oil of all trees were 2,4,6-trimethoxy-3-methylacetophenone and 6-hydroxy-2,4-dimethoxy-3-methyl-acetophenone = bancroftinone (5), shown in Figure 3. This latter novel compound is related in ring substitution to isobaeckeol [38]. In one cultivated tree of unknown origin, it accounted for 85% of the leaf oil, but in natural stands it accounted for trace–3%. 2,4,6-Trimethoxy-3-methylacetophenone accounted for 23% of the oil in one tree, but usually was present in the range 0.1–3.9%.

A list of the compounds identified in the oils of this species is given in Table 4. Compounds identified at the level of formulae only have been omitted, but a complete list can be found in [29]

### 2.5. Backhousia citriodora F.Muell.

*Backhousia citriodora* F.Muell. is a small–medium sized rainforest tree, endemic to Queensland, Australia. It occurs in the Sunshine coast region of Queensland near Eumondi, Maroochydore, Noosa and Woondon, in the ranges west of Mirriamvale, in the Mackay, Whitsunday, Townsville regions, and near Herberton, Queensland [25]. Several populations have been reduced to isolated trees through land clearing.

The leaf oil of *B. citriodora* was first described by the firm of Schimmel & Co of Miltitz, Germany in 1888 [3]. It was reported to be almost entirely (95%) citral. This was confirmed in 1923 by Penfold [22]. It has since become the source of a commercial industry for supply of geranial/neral. This is detailed in a recent comprehensive review in a sister publication by Southwell [26]. The presence of (*Z*)-iso-citral (11), (*E*)-iso-citral (12) and exo-isocitral (10) in these oils has been confirmed by Doimo [39]. The oil yield is 1.1–3.2% (*w*/*w* fresh leaf).

In 1950, Mr. J. R. Archbold, who was collecting from natural stands of the species near Miriam Vale, about 300 km north west of Maryborough, QLD, noticed slight differences in the odour of the oil produced by some trees in the area, indicating that a different type of oil was being produced by some plants in the area. An examination of the oils from single tree sampled by Penfold et al., indicated that the oil from these trees, which were morphologically indistinguishable from other *Backhousia citriodora* trees, contained-L citronellal (62–80%) [23,24,25]. The trees in question were found scattered throughout a rocky hillside area of about 2 hectares. The variant trees were located in 2 pockets, each containing about 12 trees, of which about half were the variants (one tree being about 27 m in height and slightly over 2 m in girth at breast height). Nothing else was published on this citronellal variant for about the next 50 years.

As part of a systematic breeding project to produce clones of *B. citriodora* with greater percentage of citral, 16 open pollinated families were selected. From this trial, 3 trees, out of 272 sampled, gave the L-citronellal oils. As part of this trial, a re-examination of the parent trees in the population at Noosa, QLD, from which the parent trees of the 3 citronellal producing offspring had originally been obtained, was undertaken. It was found that 1 tree was producing the L-citronellal oil. Breeding trials from this 1 tree were then undertaken [25].

The composition of the leaf oils from both chemotypes are given in Table 5. This is based on the oil composition obtained from the 3 clones taken from the open pollenated trees in the breading trials for the L-citronellal chemotype and from commercially harvested material of the citral chemotype [23,26]. The oil yield from the L-citronellal chemotype was 1.8–3.2% (*w*/*w* dry weight). The structures of the numbered compounds are given in Figure 3.

### 2.6. Backhousia enata A.J.Ford

*Backhousia enata* A.J.Ford is a relatively recently described species [40,41]. It is a large shrub or tree growing to 5–15 m in height, with a trunk diameter up to 20 cm diameter at breast height. It occurs in northeastern Queensland, where it is endemic to the ‘Wet Tropics’ and is currently confined to the Tully River catchment area. It inhabits notophyll vine forest/rainforest on soil derived from rhyolite and basalt. In 2007, there were less than 200 individuals known.

The leaf oils of *B. enata*, isolated in 0.3–0.7% (*w*/*w* dry weight) were dominated by monoterpenes (see Table 6). The principal components detected were the hydrocarbons α-pinene (14–17%), and β-pinene (36–42%), accounting for approximately 80% of the leaf oils. There were lesser amounts of terpinene-4-ol (5–8%), with p-cymene, α-terpineol and myrtenol, all in similar amounts (2–5%). The major sesquiterpene detected was spathulenol (3–5%), with caryophyllene oxide, epi-cubenol, and trans-calamenene being the next most significant compounds, all at <1.5%.

The leaf oil of *B. enata* bears no similarity to that of *B. myrtifolia*, its nearest morphological relative, whose leaf oil is dominated by the aromatic ethers, elemicin, isoelemicin, methyl eugenol or methyl isoeugenol.

### 2.7. Backhousia hughesii C.T.White

*Backhousia hughesii* C.T.White is a tree growing up to 30 m in height. It grows in the Atherton tablelands (in the Cook pastoral district) of Queensland [33]. Early work on the leaf oil of this species by Jones and Lahey, published in 1938 [7], showed that it contained mostly D-α-pinene and D-β-pinene. Brophy et al. [29] in 1995, who examined the oil of this species from 3 populations, found that the oil contained mainly sesquiterpenes rather than monoterpenes. One tree contained 12% of α-pinene, but all others examined contained <5%. The oil yield (on a fresh weight basis) was 0.13–0.45%. In contrast to other *Backhousia species*, there appeared to be only one chemotype.

The major components found were β-bisabolene (1–44%) and β-selinene (8–54%). These two components were accompanied by lesser amounts of α-copaene (0.1–4.0%), β-elemene and a bergamotene (isomer not identified) (4–20%), β-caryophyllene (0.7–4.0%), aromadendrene (0.5–7.0%), allo-aromadendrene (0.7–2.0%), E-β-farnesene (0.1–2.9%), viridiflorene (0.5–4.6%) and δ-cadinene (1.0–7.0%). The major oxygenated sesquiterpenes were caryophyllene oxide (0.4–5.0%, 1 tree 21%), globulol (0.3–9.0%), viridiflorol (0.2–3.2%) and spathulenol (0.9–2.1%). There were numerous unidentified oxygenated sesquiterpenes in the range (0.1–1.3%). A list of identified compounds found in this species is given in Table 7, compounds identified at the level of formulae only have been omitted here, but are listed in [29]. No other work has been published on this species.

### 2.8. Backhousia kingii Guymer

*Backhousia kingii* Guymer is a relatively recently described species [30]. It is a tree growing up to 20 m and is endemic to subcoastal, central eastern Queensland in the Leichhardt, Wide Bay and Burnett pastoral districts [33]. It grows in noto- or microphyll semi evergreen vine thickets in an altitude range of 0–400 m above sea level.

Only one chemotype was found in the leaf oils of *B. kingii*, which was obtained in 0.2–0.7% yield (fresh weight basis) from collecting sites. The leaf oil was essentially monoterpenoid in character (Table 8), with the principal components being α-pinene (24–49%, the majority being towards the higher value), limonene (7–24%) and 1,8-cineole (10–17%). The usually detected monoterpene hydrocarbons were present but in amounts of <0.5%. Of the oxygenated monoterpene, the major contributor was α-terpineol (4–10%), and this was accompanied by numerous other compounds (mostly <0.5%), including α-campholenic aldehyde, camphor, linalool (0.5–1.0%), terpinene-4-ol, borneol (1–2%) and *cis*-p-mentha-1,8-dien-6-ol.

The sesquiterpenes, while numerous, were not abundant in quantity. The major members were the alcohols E-nerolidol (0.2–2.8%), globulol (1.9–2.2%) viridiflorol (0.2–0.3%) spathulenol (0.1–0.3%) and α-cadinol (0.2–0.5%). The major sesquiterpene hydrocarbons were viridiflorene (0.3–0.4%) and α-bulnesene (0.1–0.2%), other sesquiterpene hydrocarbons were usually in amounts about one tenth of these values. Compounds identified at only formula level have not been included in the following table, but can be found in [29].

### 2.9. Backhousia leptopetala (F.Muell.) M.G.Harr.

As a result of phylogenetic analyses performed on a combined chloroplast and nuclear dataset of all species of *Backhousia*, Harrington et al. [1] determined that the two species of *Choricarpia* belonged in the genus *Backhousia*. These two species have previously had their essential oils analysed in 1994, as *Choricarpia* [31], and the analyses are given here for these species as members of the genus *Backhousia.*

*Backhousia leptopetala* (F.Muell.) M.G.Harr., which grows to a height of 20 m, occurs over a range from Buderim in south-east Queensland to Stanwell park south of Sydney, NSW. The leaf oil of this species, obtained from trees at 4 sites, showed an oil dominated by monoterpenes (see Table 9). The principal component was α-pinene (50–66%, one tree 29%), and this compound was accompanied by lesser amounts of limonene (1–2%, one tree 23%), p-cymene (1–13%) and 1,8-cineole (4–20%), the majority of trees being in the lower ranges. Oxygenated monoterpenes were present and the major members were pinocarvone (0.5–4%), *trans*-pinocarveol (3–14%, terpinene-4-ol (1–6%) and α-terpineol (2–4%).

Sesquiterpenes were present in small amounts (<10%). The major members were aromadendrene (0.2–0.6%), globulol (0.2–3%), viridiflorol (0.1–1%), spathulenol (0.1–0.4%) and α-eudesmol (0.3–2%). Small amounts of the aromatic ethers, methyl eugenol (0.10–3%), *E*-methyl isoeugenol (0.1–0.4%) and elemicin (0.2–2%) were also detected.

### 2.10. Backhousia myrtifolia Hook. & Harv.

*Backhousia myrtifolia* Hook. & Harv. is a shrub to tree which grows to 30 m in height, found in watercourses in coastal rainforests from Bega (New South Wales) to Miriam Vale in Queensland [32,42]. Early work by Penfold et al. in 1922 [4] showed that the leaf oil from plants growing at Lane Cove and Currowan, NSW, contained elemicin (14), (75–80%), α-pinene and some unidentified compounds. Later work by Penfold et al. in 1953 [5] and Hellyer et al. [6] showed the existence of three more chemical varieties in *B. myrtifolia*: with isoelemicin (13), methyl eugenol (16) and methyl isoeugenol (15) forms, giving a total of four chemotypes in all.

As part of the survey of the oils of *Backhousia*, Brophy et al. were able to confirm the presence of the three chemotypes (elemicin, methyl eugenol, and methyl isoeugenol) [29], but in the trees available were not able to confirm the isoelemicin chemotype, first recorded by Penfold in 1953, in the trees they examined.

The analyses of samples of the methyl eugenol methyl isoeugenol, and elemicin chemotypes, together with the isoeugenol chemotype, taken from Penfold et al. [5] are listed in Table 10. The oil yields obtained by Brophy et al. in 1995 [29] were in the range 1.0–2.2% (on fresh weight basis), although 1 tree of the elemicin chemotype gave a yield of 0.5%. The oil yields obtained by Penfold et al. and Hellyer et al. were lower, despite the fact that they were measured on a dry weight basis (0.1–0.7%).

As can be seen from Table 10, one aromatic ether dominated the oil from each chemotype. The compound is accompanied by a large number of terpenes (usually sesquiterpenes). Several compounds that were identified only at the formula level have not been included here, but can be found in [29]. Structures of numbered compounds are given in Figure 3.

### 2.11. Backhousia oligantha A.R.Bean

*Backhousia oligantha* A.R.Bean, called Backhousia sp. (Dicot Pilferer 12671) in a previous publication [29], is a small tree growing to a height of 4 m, but is often multi-stemmed, forming a low groundcover. It is found in semi-evergreen microphyll vine thicket near Biggenden in the Wide Bay pastoral district of south-east Queensland [29,43].

The leaf oil obtained from *B. oligantha* in 0.3% yield (based on dry weight of leaves) was a mixture of terpenes and alkanols and alkyl esters (see Table 11). In this respect, it resembles the oil from *B. bancroftii*. The major terpenes detected in the oil were α-pinene (11%), and β-caryophyllene (12%). There were lesser amounts of β-pinene (5%), limonene (4.3%), bicyclogermacrene (3%), α-, β- and γ-eudesmols (2.8%, 2.9% and 3.6%, respectively). There were a large number of both mono- and sesquiterpenes present in small amounts (<1%).

The leaf oil also contained a homologous series of both alkanols and their corresponding acetates. The series commenced octanol (0.2%) and continued to tetradecanol (0.4%), with the principal members being decanol (2.2%) and dodecanol (8.2%). The alkyl acetates were present, with the odd numbered members being present in lesser amounts compared to the even numbered members. The alkyl acetates present corresponded to the alkanols found, the principal members being decyl acetate (1.5%) and dodecyl acetate (8.0%). Several propionate esters were also detected (decyl- and dodecyl-), but were present in amounts of less than (0.4%).

### 2.12. Backhousia sciadophora F.Muell.

*Backhousia sciadophora* F.Muell. is a tree attaining a height of 30 m, and occurs in drier rainforest gorges and steep slopes from Dungog (NSW) to Nambour (QLD) [42,43]. The oil was first reported on by Penfold in 1924 [27]. He reported that the oil from this species contained D-α-pinene (80–85%), the remainder of the oil being sesquiterpenoid.

Brophy et al. found that the oil of this species, which was obtained in 0.4–0.5% (based on fresh leaves from 2 sites) contained α-pinene (44–55%) as its major component (see Table 12). This compound was accompanied by lesser amounts of β-pinene (2.4–8%), limonene (6.5–12.7%) and camphene (1.1–2.4%), with other monoterpene hydrocarbons accounting for <0.5% each. While oxygenated monoterpenes were reasonably plentiful they did not contribute significantly to the oil, with the major contributors being α-terpineol (2.8–6.7%), linalool (1.9–2.9%), citronellol (0.6–2.0%), borneol (1.4–2.8%), fenchol (0.8–1.7%), *trans*-pinocarveol (0.2–0.7%) and α-campholenic aldehyde (0.5–0.9%).

Sesquiterpenes, though comprising almost half the number of compounds detected in the oil, accounted for <20% of the oil. The major members were alcohols, α-, β- and γ-eudesmol (each 0.8–3.3%). There were numerous other oxygenated hydrocarbons in amounts <0.5%. The major sesquiterpene hydrocarbons were δ-cadinene (2.0–2.4%), and α-copaene (0.5–1.0%). Compounds detected at only formula levels are not included in this table, but are listed in [29]. The oil of this species bears similarities to that of *B. kingii*, from which it was separated by Guymer [30]. The list of compounds identified is given in Table 12.

### 2.13. Backhousia subargentea (C.T.White) M.G.Harr.

*Backhousia subargentea* (C.T.White) M.G.Harr. is also the result of phylogenetic analyses performed on a combined chloroplast and nuclear dataset of all species of *Backhousia,* Harrington et al. [1], that determined the two species of *Choricarpia* belonged in the genus *Backhousia*. These two species have previously had their essential oils analysed in 1994, as *Choricarpia* [31], and the analyses are given here for these species as members of the genus *Backhousia*.

*Backhousia subargentea,* examined from seven sites, produced a monoterpenoid leaf oil. The principal components (see Table 13) were α-pinene (30–76%), limonene (2–55%), and 1,8-cineole (2–20%). Other regularly encountered hydrocarbons accounted for <5%. Oxygenated monoterpenes were the next most significant group, with pinocarvone (0.5–3%), *trans*-pinocarveol (2–14%), α-terpineol (0.5–3%) and a mixture of p-menthadienols (0.1–1%) accompanying the already mentioned 1,8-cineole. This latter group of compounds, which formally could arise from autoxidation of limonene in the plant, has often been observed in the essential oils of eucalypts, but are much rarer in the melaleucas (Brophy, unpublished). Isoamyl-isovalerate (0.6–7%) was also present in the oil.

Sesquiterpenes accounted for less than 10% of the oil despite their significant numbers. The major members of this group were globulol (1–3%), with viridiflorol, α-, β-, and γ-eudesmol each being <3%. The sesquiterpene hydrocarbons were minor contributors, with aromadendrene (9–12%) being the largest contributor. Of significance, in this leaf oil, was the presence of jensenone (4,6-diformyl-2-isopentanoylphloroglucinol), whose structure is listed in Figure 3 (17), present in all samples at levels of <5%. This compound was first encountered in the leaf oil of *Eucalyptus jensenii*, where it accounted for over 50% of the steam volatiles [44]. The list of compounds identified is given in Table 13.

### 2.14. Syzygium anisatum (Vickery) Craven & Biffin

*Syzygium anisatum* (Vickery) Craven & Biffin (syn *Backhousia anisata*) is a fairly dense glabrous foliage tree that can reach 50 m in height and have a circumference of 4 m. It inhabits rainforests in a few places in the Bellingen and Nambucca valleys of northern New South Wales [42]. In its natural state, it is regarded as a rare and endangered species [16,45]. The species, since then, has had two changes of name as its taxonomy has been reinvestigated, passing through *Anetholea anisata* (Vickery) Peter G., Wilson [20], and finally being placed in *Syzygium anisatum* (Vickery) Craven & Biffin [19]. Due to its long history in *Backhousia*, its leaf oils are still considered here in this review.

McKern was the first to analyse the oil of *B. anisata* and found it to contain anethole at about 60% of the oil, the oil yield being 0.5% [15]. Brophy and Boland [16] reported that two chemotypes existed, having an oil yield of 1.3–2.0% (*w*/*w* fresh leaf) for both chemotypes of this species. The methyl chavicol (18) chemotype was found in approximately 25% of the trees examined (9 trees, including 1 bulk of 3 trees). Blewitt and Southwell [18], in a later and more widespread survey, found that the methyl chavicol (18) chemotype was approximately 1: 4.7 of the *E*-anethole (19) chemotype. They found that three of the ten sites sampled contained both chemotypes occurring within meters of each other. Southwell et al. also found that a few trees contained approx. equal amounts of both *E*-anethole and methyl chavicol [17].

Brophy and Boland reported that the percentage of E-anethole was 93–95% in the trees examined (*n* = 10) [16]; Blewitt and Southwell [18] found 71.2–93.7% in a larger sample (*n* = 17). In this chemotype, the methyl chavicol percentage was 4.4–5.6% [16] and 5–15.3% [18]. For the second chemotype the methyl chavicol percentage was 66–73% [16] and 55.98–75% [18]. In this second chemotype, the percentage of *E*-anethole was 20–33% [16] and 22.1–42.8% [18]. Terpenes were of very minor importance in both oils [16]. The structures of E-anethole and Methyl chavicol are given in Figure 3, while the compounds identified in the oils are given in Table 14.

## 3. Discussion

In their 2012 paper [1], Harrington et al. argued that “there were four strongly supported clades containing two to four taxa, with no support for relationships among clades, and the relationships of *Backhousia bancroftii* and *B. citriodora* remain unresolved”. They also state that on the analyses of the DNA data “The current distribution of *Backhousia* is inferred to be largely due to the contraction of Australian rainforests in the Neogene”. This is supported by Figure 2 in their paper [1].

From this diagram, it might be expected that species grouped together might have similar leaf oils, and that the closer together the species were grouped, the more similar the leaf oils of the species might be.

**Figure 4:**
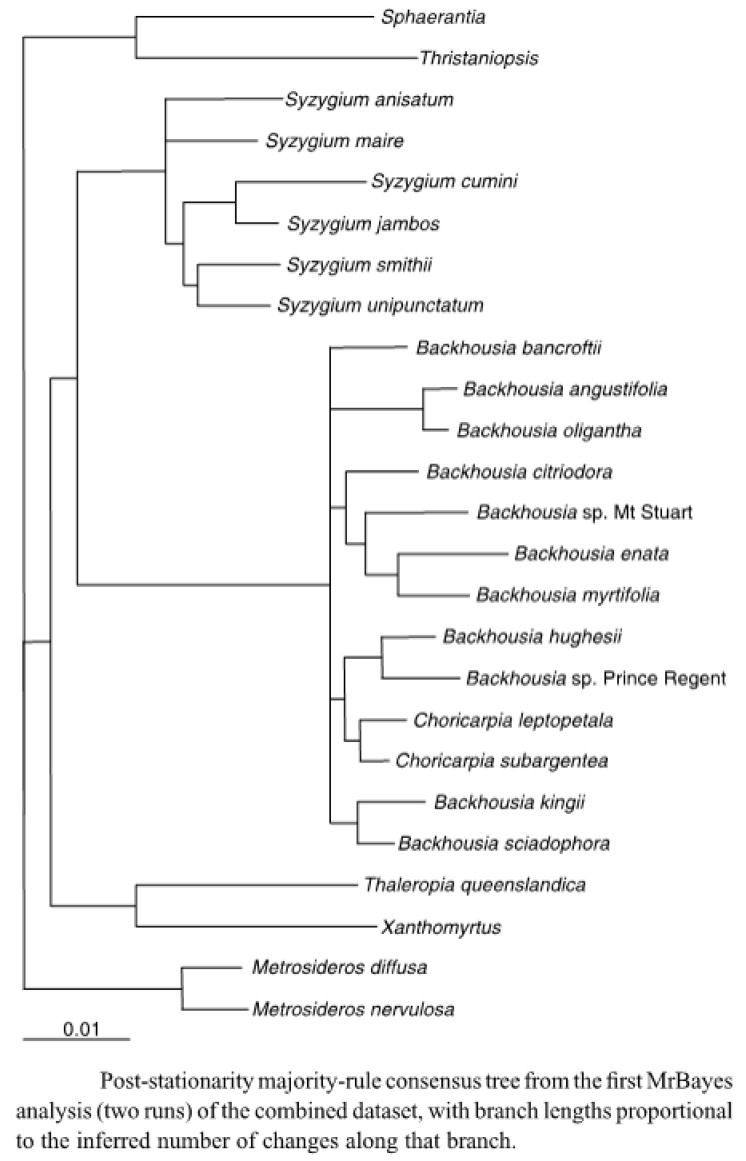
Dendrogram of Figure 2 from the 2012 paper by Harrington et al. [1]. Used with permission of *Australian Systematic Botany*.

Examining the dendrogram, Figure 4, there appear to be a significant number of species where their close proximity is also reflected in the leaf oils. Thus *B. leptopetala* and *B. subargentea*, species that have been transferred from the genus *Choricarpia*, (and in the dendrogram are still mentioned as species of *Choricarpia*) do possess similar leaf oils, which are heavily based on monoterpenes, with α-pinene, limonene, and 1,8-cineole being prominent compounds in both species. There are, however, other compounds, present in small amounts, that do differ between the species.

*Backhousia kingii* was relatively recently split form *B. sciadophora* [30]. Both species possess similar leaf oils, in which monoterpenes predominate, with α-pinene and limonene being prominent components and sesquiterpenes being only minor components.

*Backhousia hughesii* and *B. gundarara* do not, however, follow this line, with *B. hughesii* having an oil rich in sesquiterpenes, with β-elemene and β-bisabolene being the major components. *B. gundarara*, (*Backhousia* sp. Prince Regent in Figure 4) while possessing major amounts of globulol, viridiflorol, spathulenol and other sesquiterpene hydrocarbons, also contains considerable amounts of α-pinene, limonene and other monoterpene hydrocarbons. It also contains a series of, as yet, unidentified aromatic compounds, whose mass spectra are given in the footnote to Table 1.

Of the three species in the clade containing *B. myrtifolia*, *B. enata* and *B. tetraptera*, *B. myrtifolia* stands out distinctively because of the presence of the aromatic ethers, methyl eugenol, *E*-methyl isoeugenol, elemicin and *E*-isoelemicin, as a principal component in its leaf oil, vastly overshadowing any other terpenoid components. The other two species contain mainly monoterpenoid leaf oils, with the *B. enata* oil being dominated by α-pinene and sabinene, while in the case of *B. tetraptera* (*Backhousia* sp. Mt. Stuart in Figure 4), the major components were myrtenyl acetate and linalool. *B. citriodora*, whose leaf oil is dominated by either citral or L-citronellal, stands apart from the other members of this clade.

In the clade containing *B. bancroftii*, *B. angustifolia* and *B. oligantha*, *B. bancroftii* has an “unresolved” morphological relationship to the other two species [1], but the contents of its leaf oil, containing major amounts of alkyl acetates and alcohols, is a lot more closely related to the oils of *B. oligantha*, which also contains significant amounts of these compounds. These two species are the only species of *Backhousia* to contain the alkyl esters and alcohols in any quantity. *B. bancroftii* also contains varying amounts (trace to 23%) of 2,4,6-trimethoxy-3-methylacetophenone and bancroftinone (5) (trace—>80%), not present in any other species of *Backhousia*.

*Backhousia angustifolia*, on the other hand, presents several chemotypes whose leaf oils, apart from containing significant amounts of α-pinene and 1,8-cineole, could also contain the benzochromone, angustifolionol (1), or the β-triketones angustione (2), and/or dehydroangustione (3), or the ketone angustifolenone (4), none of which occurred in either *B. oligantha* or *B. bancroftii*.

## 4. Conclusions

The relationship of the leaf oils of a species of *Backhousia* to that species’ place in the dendrogram (Figure 4) is rather problematic. Two species (*B. bancroftii*, *B. oligantha*) possess similar oils, containing a series of alkanols and their corresponding acetate esters, rare in the oils of *Backhousia*, though *B. bancroftii* bears an ’unresolved’ relationship to *B. oligantha.* In other cases, e.g., *B. kingii*, *B. sciadophora*, the leaf oils are very similar and, in fact, *B. kingii* was split from *B. sciadophora* on morphological grounds. The two species which, in terms of classes of compounds, are most similar, *B. myrtifolia*, containing di- or tri-methoxy-allyl or -propenyl benzene, and *Syzygium anisatum*, containing methoxy-allyl or-propenyl benzene, are no longer in the same genus. It would appear that with our present knowledge, it would be wise to not place too much reliance on the relative grouping of the species when considering their leaf oils: more research on the genes directing the syntheses of these components is required.

## 5. Materials and Methods

*Isolation of oils*: The leaf oils were isolated by hydrodistillation with cohobation as previously outlined in [46]. Analyses of the oils were carried out by gas chromatography and combined gas chromatography-mass spectrometry. The oil yields quoted are weight/weight, based on fresh material.

*Plant material:* Leaves of *Backhousia gundarara* were obtained from a cultivated plant, grown at Tolga, QLD. The plant originated from the Kimberly region, WA., voucher: Caroline Range, G. & N. Sankowsly *Sanko 2255*, 16 September 2003 (PERTH), CNS 136982.1. *B. tetraptera* was from 4 individual trees growing at Mount Stuart, Townsville, QLD, and a cultivated tree growing at Tolga, QLD, originally from Mt. Stuart, voucher: J.W. Elliott JE10 & K. Townsend, 21 October 2010 (CNS).

Analyses of the oils were by combined gas chromatography/mass spectrometry (GC/MS) and gas chromatography with compounds identified by their mass spectra, GC retention time and Linear Retention Indices (LRI) relative to n-alkanes [47,48] and by comparison of their mass spectra with either known compounds or published spectra [47]. Analytical gas chromatography (GC) was carried out on a Shimadzu GC17A gas chromatograph (Kyoto, Japan) on a BP-20 column (60 m × 0.25 mm × 0.25 µm), which was programmed from 50–220 °C at 3 °C/min with helium as the carrier gas. Injector and detector were both 220 °C. GC integrations were performed on a SMAD electronic integrator (Morgan Kennedy). GCMS was carried out on a Shimadzu GCMS-2020 mass spectrometer operating at 70 eV ionization energy (GC column used was a BP-20 (30 m × 0.25 mm × 0.25 µm) programmed from 35 to 220 °C at 3 °C/min with helium as the carrier gas, injector temperature was 250 °C). Some analyses were also carried out by GCMS on a DB-5 column (30 m × 0.25 mm × 0.25 µm) programmed from 35 to 250 °C at 5 °C/min, with helium as carrier gas and mass spectrometer conditions as for the previous column. Mass spectra were recorded in electron impact (EI) mode at 70 eV, scanning the 41–450 *m*/*z* range. Interface and source temperatures were 250 °C and 220 °C, respectively, with 1 scan/sec cycle time.

*Literature searches*: Literature searches were performed using SciFinder Scholar using appropriate key words for all species. Where possible, original literature was then cited.

## Figures and Tables

**Table 1:** Components identified in the leaf oil of *Backhousia gundarara*.

	Linear Retention Index		Linear Retention Index		
	BP20		BP5		
Compound	Found	Literature [32]	Found	Literature [32]	%
α-pinene	1010	1025	936	936	13.6
α-thujene	1016	1027	934	928	0.2
camphene	1068	1068	957	950	tr
β-pinene	1109	1110	981	978	1.4
Sabinene	1109	1122	976	973	0.2
α-phellandrene	1192	1168	1010	1004	tr
p-menth-1(7),8-diene	1158	1154		1006	0.2
Myrcene	1158	1161	993	989	0.5
1,4-cineole	1165	1186	1020	1017	0.1
Limonene	1183	1198	1034	1029	3.6
β-phellandrene	1197	1209	1036	1030	0.7
1,8-cineole	1191	1211	1038	1032	0.7
γ-terpinene	1232	1245	1064	1060	tr
p-cymene	1256	1270	1031	1024	1.2
terpinolene	1269	1282	1090	0087	tr
α-pinene oxide	1352	1363	1090	1087	tr
α-cubebene	1450	1460			0.1
δ-elemene	1456	1468	1341	1337	0.3
bicycloelemene	1468	1487	1337	1333	0.1
Linalool	1551	1543	1100	1099	0.2
*trans*-menth-2-en-1-ol	1557	1584	1153	1138	0.1
β-elemene	1573	1590	1396	1390	2.0
β-caryophyllene	1582	1598	1430	1420	2.7
terpinen-4-ol	1593	1601	1192	1177	1.5
aromadendrene	1649	1620	1451	1441	1.6
Cryptone	1642	1672	1204	1184	0.2
α-humulene	1643	1667	1468	1453	0.3
C_15_H_24_, unknown **X**	1673				2.1
viridiflorene	1687	1696	1803	1492	1.2
α-terpineol	1691	1694	1208	1190	0.8
β-selinene	1692	1717		1486	0.4
α-selinene	1698	1725		1493	0.5
bicyclogermacrene	1709	1735	1509	1494	1.4
germacrene-A	1736	1747	1521	1518	0.6
palustrol	1904	1930	1587	1567	0.6
ledol	2006	2039	1570	1567	0.7
cubeban-11-ol	2037	2074	1613	1588	1.4
globulol	2057	2082	1604	1582	6.1
viridiflorol	2066	2090	1613	1591	3.3
rosifoliol ..tent..	2089			1600	1.2
spathulenol	2109	2127	1594	1576	11.1
unknown	2126				0.4
2,4,6-trimethoxytoluene	2161		1497	1486	0.4
Selina-11-en-4-ol	2233	2252			1.2
Compound **A**, MW220			1831		0.6
Compound **B**, MW 264	>2500		1852		2.6
Compound **C**, MW 252	>2500		1848		0.4
Compound **D**, MW 250	>2500		1885		0.2
Compound **E**, MW 250	>2500		1911		1.7
Compound **F**, MW 236	>2500		1936		0.6
Compound **G**, MW 234	>2500		2040		3.0
Compound **H**, MW 220	>2500		2026		0.3

Mass spectra of unidentified compounds. **A**: *m*/*z* (%) 220 (M+, 10%), 202 (12), 195 (60), 177 (70), 164 (30), 149 (55), 137 (70), 119 (35), 107 (33), 99 (30), 91 (50), 81 (35), 55 (38), 43 (100). **B**: *m*/*z* (%) 264 (M+, 85%), 249 (45), 247 (46), 235 (33), 223 (100), 216 (25), 209 (73), 193 (30), 165 (25), 91 (20), 69 (48), 41 (35). **C**: *m*/*z* (%) 252 (M+, 10%), 209 (100), 194 (8), 166 (3), 151 (3), 136 (7). **D**: *m*/*z* (%) 250 (M+, 56%), 228 (15), 219 (63), 209 (100), 204 (20), 194 (28), 182 (38), 165 (12), 151 (33), 136 (20), 133 (17), 105 (12), 91 (24), 77 (20), 53 (10), 45 (45). **E**: *m*/*z* (%) 250 (M+, 50), 235 (40), 233 (35), 221 (20), 209 (100), 195 (40), 179 (8), 165 (6), 151 (15), 136 (15), 121 (5), 106 (7), 91 (12), 69 (25), 41 (25). **F**: *m*/*z* (%) 236 (M+, 100), 221 (55), 218 (27), 207 (35), 194 (450), 190 (30), 179 (35), 166 (45), 165 (40), 151 (75), 123 (18), 95 (22), 77 (15), 69 (25). **G**: *m*/*z* (%) 234 (M+, 100), 219 (95), 213 (26), 189 (12), 161 (8), 151 (10), 135 (5), 95 (7), 77 (8), 55 (10), 43 (15). **H**: *m*/*z* (%) 220 (M+, 100) 219 (95), 205 (15), 202 (19), 191 (42), 189 (30), 161 (8), 149 (5), 133 (13), 102 (7), 73 (10), 69 (17), 68 (15), 55 (18), 43 (20). **X**: LRI 1673 (BP20) *m*/*z* (%) 204 (M+, 7%), 189 (15), 175 (5), 161 (25), 147 (5), 135 (12), 119 (45), 105 (35), 91 (100), 79 (30), 55 (20), 43 (44).

**Table 2:** Components identified in the leaf oil of *Backhousia tetraptera*.

	Linear Retention Index		Linear Retention Index		
	BP20		BP5		
Compound	Found	Literature [32]	Found	Literature [32]	%
α-pinene	1005	1025	936	936	3.7–3.8
β-pinene	1080	1110	974	978	0.7–1.0
sabinene	1100	1122	978	973	tr
myrcene	1150	1161	989	989	0.1
α-terpinene	1155	1178	1014	1017	tr
limonene	1174	1198	1025	1030	0.2–0.3
1,8-cineole	1179	1211	1028	1031	tr
β-terpinene	1221	1245	1057	1060	0.1
*E*-β-ocimene	1236	1250	1049	1048	tr-0.2
p-cymene	1244	1270	1025	1024	0.1
terpinolene	1256	1282	1084	1087	0.1
6-methyl-hept-5-en-2-one	1320	1337		986	tr
hexanol	1340	1351		869	0.1
*cis*-linalool oxide (furanoid form)	1420	1446	1075	1075	0.1
δ-elemene	1444	1469	1339	1337	tr-0.4
*trans*-linalool oxide (furanoid form)	1454	1454	1087	1083	0.1
α-copaene	1450	1491	1377	1376	0.1–0.3
campholenal	1460	1496	1123	1124	tr-0.1
pinocarvone	1525	1576	1160	1161	0.1–0.3
linalool	1539	1543	1100	1099	5.0–8.9
hotrienol	1591	1602	1104	1104	0.2
β-elemene	1560	1590	1393	1390	0.3–0.8
β-caryophyllene	1557	1598	1421	1420	1.4–5.0
aromadendrene	1573	1620	1441	1441	0.3–0.7
terpinen-4-ol	1577	1601	1176	1177	0.1–0.3
myrtenal	1584	1632	1194	1192	0.1–0.3
allo-aromadendrene	1601	1649	1463	1460	0.7–1.4
*trans*-pinocarvyl acetate	1623	1638	1298	1295	0.2–0.3
*trans*-pinocarveol	1625	1661	1137	1140	tr-0.1
α-humulene	1627	1667	1456	1453	0.8–2.3
myrtenyl acetate	1658	1692	1326	1329	24.1–40.5
germacrene-D	1669	1708	1480	1481	0.3–0.9
α-terpineol	1675	1694	1489	1492	0.3–0.7
viridiflorene	1675	1696	1189	1192	1.98–6.3
α-selinene	1685	1725	1498	1493	0.7–2.3
bicyclogermacrene	1692	1735	1493	1494	0.1–1.1
*E,E*-α-farnesene	1732	1744	1509	1504	0.1–1.6
mentha-1,5-dien-8-ol	1706	1674	1165	1167	tr-0.2
myrtenol	1763	1790	1195	1194	0.5–2.1
*trans*-carveol	1812	1836	1220	1217	0.1
palustrol	1884	1930	1570	1567	tr
caryophyllene oxide	1929	1986	1586	1581	2.6–3.7
epi-globulol	1998	2100	1588	1585	0.1–0.5
ledol	2030	2039	1560	1567	0.3
humulene epoxide II	1986	2047	1612	1605	1.2–1.6
methyl eugenol	1992	2063	1401	1402	tr-0.2
cubeban-11-ol	2015	2074	1588	1588	0.1
globulol	2036	2082	1584	1582	0.8–1.4
viridiflorol	2044	2090	1594	1591	0.3–0.7
spathulenol	2088	2127	1780	1576	7.0–8.5
eugenol	2132	2163	1358	1358	0.9–1.2
2,4,6-trimethoxytoluene, mw 182	2146		1494	1486	0.6–0.9

**Table 3:** Compounds identified in four trees of *Backhousia angustifolia*.

Compound	Tree 1	Tree 2	Tree 3	Tree 4
	%	%	%	%
α-thujene	0.02	0.04	0.04	tr
α-pinene	1.7	4.6	0.04	0.7
camphene	tr	tr	0.01	tr
β-pinene	0.2	0.1	0.5	0.5
sabinene	0.5	1.2	0.1	tr
myrcene	0.2	0.1	0.3	
α-phellandrene	0.03	0.02	0.1	
α-terpinene	0.03	0.02	0.1	
limonene	0.02	tr	1.7	0.7
1,8-cineole	13.7	32.1	15.3	6.7
*Z*-β-ocimene	tr	tr	0.01	
*E*-β-ocimene	0.9	0.1	13.2	
p-cymene	0.03	0.1	0.1	
α,p-dimethylstyrene	tr	tr	0.1	
angustifolenone	tr	11.7	0.4	0.1
terpinene-4-ol	0.2	0.2	0.4	0.1
β-caryophyllene	0.1	0.2	0.2	
aromadendrene	0.1	0.02	0.2	
α-bulnesene	tr	tr	0.03	
α-humulene	tr	tr	0.2	
δ-terpineol	tr	tr	0.1	
α-terpineol	1.5	3.2	1.6	0.5
viridiflorene	tr	tr	0.3	
bicyclogermacrene	1.1	0.1	0.5	
globulol	0.1	0.04	0.6	
viridiflorol	0.1	0.04	0.3	
spathulenol	0.2	0.2	0.6	
dehydroangustione	tr	24.1	tr	85.0
angustione	75.8	23.4	60.0	5.0
angustifolionol	2.0			5.0
platyphyllol	<1	<1	<1	<1

**Table 4:** Compounds identified in the leaf oil of *Backhousia bancroftii*.

Compound	%	Compound	%
α-pinene	0–6.2	bicyclogermacrene	tr-1.1
camphene	0-tr	*E,E*,-α-farnesene	tr-0.4
β-pinene	0–1.3	δ-cadinene	tr-1.8
limonene	0–0.1	decanol	0.1–17.4
β-phellandrene	0-tr	cadina-1,4-diene	0–0.2
1,8-cineole	0–0.2	decyl butyrate	tr
hexyl acetate	0-tr	calamenene	tr-0.4
3-hexenol	0-tr	benzyl alcohol	tr-0.1
methyl octanoate	0–0.1	dodecyl acetate	0.2–21.0
α-cubebene	0-tr	a calacorene	0-tr
octyl acetate	0.3–61.7	palustrol	tr
bicycloelemene	0-tr	dodecan-1-ol	tr-23.0
α-copaene	0–3.2	caryophyllene oxide	tr-0.6
octyl propanoate	0–0.1	cubenol	0–0.1
octan-1-ol	0.1–2.2	epi-cubenol	0–0.1
nonyl acetate	0–0.5	cubeban-11-ol	0–0.4
β-elemene	0–0.1	globulol	tr-1
β-caryophyllene	Tr-3	viridiflorol	tr-1
terpinene-4-ol	0–0.4	tetradecyl acetate	tr-1.8
aromadendrene	0–4.2	spathulenol	tr-2.7
α-bulnesene	0-tr	T-muurolol	0-tr
octyl butyrate	0–0.1	δ-cadinol	0-tr
allo-aromadendrene	0–1.6	unknown, mw 178	tr
decanal	0–0.1	unknown, mw262	tr
α-humulene	0–2.4	unknown, mw 148	tr
decyl acetate	0.5–39.0	2,4,6-trimethoxy-3-methylacetophenone	tr-23.0
viridiflorene	tr	bancroftinone	tr-84.6
α-terpineol	0–0.2		

**Table 5:** Composition of the leaf oils of the two chemotypes of *Backhousia citriodora*.

Compound	L-Citronellal Form %	Citral Form %
myrcene	0.3	tr–0.7
2,3-dehydro-1,8-cineol (6)		tr–0.9
6-methyl- 5-hepten-2-one		tr–2.9
L-citronellal (7)	85–89	tr–1.0
linalool	0.3–0.4	tr–1.0
isopulegol isomer	1.6–2.5	
isopulegol isomer	4.0–6.4	
isopulegol isomer	0.2–0.3	
neral (8)	0.2–0.5	32.0–40.9
geranial (9)	0.2–0.6	44.0–60.7
exo-isocitral (10)		tr–2.0
*Z*-isocitral (11)		tr–2.7
*E*-isocitral (12)		tr–4,3
nerol		tr–0.6
citronellol	2.6–3.2	
geraniol	0.1–0.2	0.5–2.5

**Table 6:** Compounds identified in the leaf oil of *Backhousia enata*.

Compound	%	Compound	%
α-pinene	14.9	*trans*-pinocarveol	2.6
α-fenchene	tr	p-menth-1,5-dien-8-ol	0.3
camphene	0.1	α-terpineol	2.9
β-pinene	36.2	δ-cadinene	1.1
sabinene	1.8	myrtenol	2.1
α-phellandrene	0.1	*trans*-calamenene	1.2
α-terpinene	0.8	*trans*-p-mentha-1,8-ien-8-ol	0.2
limonene	1.9	p-cymen-8-ol	0.2
β-phellandrene	0.3	β-calacorene	0.2
1,8-cineole	0.5	α-calacorene	0.3
γ-terpinene	1.5	caryophyllene oxide	1.4
p-cymene	4.6	humulene oxide	0.1
terpinolene	0.7	cubenol	0.8
linalool	0.4	epicubenol	1.2
pinocarvone	1.4	spathulenol	4.3
pinocamphone	0.2	T-cadinol	0.2
α-fenchol	0.3	T-muurolol	0.3
terpinene-4-ol	7.3	α-muurolol	0.5
myrtenal	1.5	α-cadinol	0.4

**Table 7:** Compounds identified in the leaf oil of *Backhousia hughesii*.

Compound	%	Compound	%
α-pinene	0.3	allo-aromadendrene	1.3
α-fenchene	tr	*E*-β-farnesene	2.9
camphene	0.1	viridiflorene	1.4
β-pinene	0.4	β-elemene	19.2
myrcene	tr	β-bisabolene	44.0
α-terpinene	tr	bicyclogermacrene	0.7
limonene	0.1	δ-cadinene	2.2
γ-terpinene	tr	cadina-1,4-diene	0.1
*E*-β-ocimene	tr	benzyl alcohol	Tr
p-cymene	tr	palustrol	0.1
terpinolene	tr	caryophyllene oxide	0.9
δ-elemene	0.1	cubenol	0.2
bicycloelemene	0.2	epi-cubenol	0.2
α-copaene	Tr	cubeban-11-ol	0.5
β-bourbonene	0.2	globulol	2.1
β-ylangene	0.1	viridiflorol	1.4
β-elemene + an α-bergamotene	3.9	spathulenol	1.1
β-caryophyllene	1.7	T-cadinol	0.2
aromadendrene	2.5	T-muurolol	0.4
α-bulnesene	0.7	δ-cadinol	0.2
		α-cadinol	0.3

**Table 8:** Compounds identified in the leaf oil of *Backhousia kingii*.

Compound	%	Compound	%
α-pinene	49.7	aromadendrene	0.03
α-fenchene	0.1	α-bulnesene	0.1
camphene	0.6	allo-aromadendrene	0.02
β-pinene	0.3	*trans*-pinocarveol	0.2
sabinene	0.1	α-humulene	0.04
myrcene	0.02	δ-terpineol	0.1
α-phellandrene	0.01	viridiflorene	0.4
α-terpinene	0.03	α-muurolene	0.5
limonene	9.6	δ-cadinene	0.6
1,8-cineole	13.8	γ-cadinene	0.5
γ-terpinene	0.5	cadina-1,4-diene	1.4
p-cymene	0.8	a bornyl ester	0.04
terpinolene	0.2	geraniol	0.1
6-methylhept-5-en-2-one	0.02	*cis*-p-mentha-1,8-dien-6-ol	0.5
fenchone	0.03	a geranyl ester	1.4
α,p-dimethylstyrene	0.02	palustrol	0.04
cis linalool oxide (furanoid)	0.02	caryophyllene oxide	0.1
*trans*-linalool oxide (furanoid)	0.02	E-nerolidol	1.2
α-copaene	0.2	cubenol	0.2
α-campholenic aldehyde	0.4	epi-cubenol	0.1
camphor	0.3	cubeban-11-ol	0.1
α-gurjunene	003	globulol	2.1
linalool	0.5	viridiflorol	0.3
pinocarvone	0.1	spathulenol	0.3
unknown, mw 182	0.3	δ-cadinol	0.3
fenchol	0.2	T-cadinol	0.3
β-caryophyllene	0.1	T-muurolol	0.1
terpinene-4-ol	0.5	α-cadinol	0.2
α-terpineol	10.0	E,E-farnesol	0.1
borneol	0.9		

**Table 9:** Compounds identified in the leaf oil of *Backhousia leptopetala*.

Compound	%	Compound	%
α-pinene	50.6	neral	0.1
α-fenchene	0.2	C_10_H_16_O	0.1
camphene	0.7	α-terpineol	3.0
β-pinene	0.2	borneol	1.4
sabinene	0.1	verbenone	0.2
C_10_H_14_	0.02	unknown, mw 254	0.6
myrcene	0.01	carvone	0.5
α-phellandrene	tr	myrtenol	0.3
α-terpinene	0.03	*trans*-mentha-1(7),8-dien-2-ol	0.3
limonene	1.7	C_10_H_16_O	0.1
1,8-cineole	7.3	*trans*-mentha-1,8-dien-6-ol	0.2
*Z*-β-ocimene	tr	p-cymene-8-ol	0.1
γ-terpinene	0.5	*cis*-mentha1,8-dien-6-ol	0.03
*E*-β-ocimene	0.1	*cis*-mentha-1(7), 8-dien-2-ol	0.2
p-cymene	2.8	unknown, mw 190	0.1
terpinolene	0.2	unknown mw 222	tr
hexanol	0.04	methyl eugenol (16)	0.1
C_10_H_16_O	0.1	*E*-nerolidol	0.03
α,p-dimethylstyrene	0.1	C_15_H_26_O	0.3
camphor	0.2	C_15_H_26_O	0.1
campholenic aldehyde	0.3	globulol	0.9
linalool	0.1	viridiflorol	0.5
pinocarvone	2.2	C_15_H_26_O	0.2
pinocamphone	0.1	C_15_H_26_O	0.2
fenchol	0.7	C_15_H_26_O	0.2
terpinene-4-yl acetate	0.2	T-muurolol	0.1
myrtenal	0.03	thymol	0.1
C_10_H_16_O	0.1	E-methyl isoeugenol (15)	0.4
C_15_H_24_	0.2	elemicin (14)	1.4
aromadendrene	0.4	carvacrol	0.1
*trans*-pinocarveol	9.2	β-eudesmol	2.2
C_15_H_24_	0.2	unknown, mw 192	0.04
		C_15_H_26_O	0.1

**Table 10:** Compounds identified in the four chemotypes of *Backhousia myrtifolia*.

Compound	Chemotype 1 %	Chemotype 2 %	Chemotype 3 %	Chemotype 4 %
α-pinene β-pinene	tr tr			
myrcene	0.02			
limonene	0.01			
*E*-β-ocimene	0.7	0.1	0.7	
terpinolene	tr			
peryllene	tr			
hexanol	0.01			
an allo-ocimene	tr			
3-hexenol	0.2	0.02	0.3	
2-hexenol	0.1		0.1	
*cis*-linalool oxide (furanoid)	0.1		0.02	
furfural	tr			
α-cubebene	tr		0.01	
*trans*-linalool oxide (furanoid)	0.1		0.01	
bicycloelemene	tr			
α-copaene	0.03	0.03	0.1	
benzaldehyde	0.1		0.02	
linalool	5.8	1.0	3.2	
β-elemene	0.04		0.02	
β-caryophyllene	0.3	0.7	0.1	
methyl benzoate	0.01		0.01	
γ-elemene	0.04		0.01	
allo-aromadendrene	0.1	0.3	0.2	
α-humulene	0.02	0.02	0.01	
viridiflorene	0.03		0.01	
α-terpineol	0.1	0.1	0.1	
β-selinene	0.2		0.1	
α-selinene	0.3		0.1	
bicyclogermacrene	0.1		0.01	
*E,E*-α-farnesene	0.3	0.2	0.1	
δ-cadinene	0.1		0.01	
methyl salicylate	0.01			
2-tridecanone	0.04		0.1	
geraniol	0.04		0.1	
a calacorene	0.1		Tr	
palustrol	0.03		Tr	
*Z*-methyl cinnamate	0.01			
caryophyllene oxide	0.1	0.02	0.01	
methyl eugenol (16)	4.9	4.0	86.4	
*E*-methyl cinnamate	0.1	0.1	0.04	
*Z*-methyl isoeugenol	1.9	0.3	0.1	
elemicin	4.6	91.5	4.1	
spathulenol	0.5	0.3	0.2	
eugenol	0.04	0.1	0.1	
*E*-methyl isoeugenol (15)	74.0	0.4	1.0	
*Z*-isoelemicin	0.1			
*E*-isoelemicin (13)	2.4		0.01	78 [5]

**Table 11:** Compounds identified in the leaf oil of *Backhousia oligantha*.

Compound	%	Compound	%
α-pinene	11.0	decyl acetate	1.5
α-fenchene	tr	viridiflorene	0.3
camphene	tr	γ muurolene	0.5
β-pinene	2.3	bicyclogermacrene	3.0
sabinene	tr	octyl propionate	tr
myrcene	0.4	a p-menthenol	0.9
α-phellandrene	0.8	δ-cadinene	1.9
α-terpinene	0.2	decanol	2.2
limonene	4.3	undecyl acetate	0.4
β-phellandrene	1.6	myrtenol	0.1
1,8-cineole	tr	calamenene	0.1
*Z*-β-ocimene	0.1	undecanol	0.5
γ-terpinene	tr	p-menth-2-en-7-ol	0.1
*E*-β-ocimene	1.8	dodecyl acetate	8.1
p-cymene	0.2	unknown, mw 190	tr
terpinolene	tr	β-calacorene	0.1
an allo-ocimene	tr	palustrol	0.1
α,p-dimethylstyrene	tr	dodecyl propionate	0.4
α-cubebene	0.1	dodecanol	8.2
bicycloelemene	tr	*E*-nerolidol	0.3
α-copaene	1.2	cubenol	0.4
octanol	0.2	epicubenol	0.2
α-bourbonene	tr	cubeban-11-ol	0.1
β-bourbonene	0.3	globulol	1.1
α-gurjunene	0.1	viridiflorol	0.7
linalool	1.5	tetradecyl acetate	0.9
pinocarvone	tr	spathulenol	1.3
β-ylangene (tent.)	0.1	γ-eudesmol	3.6
β-elemene	0.2	T-muurolol	0.9
β-caryophyllene	12.2	δ-cadinol	0.3
aromadendrene	0.2	α-eudesmol	2.8
α-bulnesene	tr	β-eudesmol	2.9
allo-aromadendrene	0.5	tetradecanol	0.4
α-humulene	1.0		

**Table 12:** Compounds identified in the leaf oil of *Backhousia sciadophora*.

Compound	%	Compound	%
α-pinene	44.2	*trans*-pinocarveol	0.2
α-fenchene	0.6	cis-piperitol	0.1
camphene	2.4	γ-muurolene	0.1
β-pinene	8.0	α-terpineol	6.7
sabinene	0.1	borneol	2.9
myrcene	tr	α-muurolene	0.6
α-phellandrene	tr	*trans*-piperitol	0.5
α-terpinene	0.1	δ-cadinene	2.0
limonene	12.7	cadina-1,4-diene	0.1
*Z*-β-ocimene	0.2	citronellol	0.6
γ-terpinene	0.7	*cis*-p-mentha-1,8-dien-6-ol	0.6
*E*-β-ocimene	tr	p-cymen-8-ol	0.1
p-cymene	0.8	α-calacorene	0.1
2-hexenol	tr	β-calacorene	0.1
fenchol	0.1	cubenol	0.1
*cis*-linalool oxide (furanoid)	0.1	epi-cubenol	0.1
*trans*-linalool oxide (furanoid)	tr	cubeban-11-ol	0.6
α-campholenic aldehyde	0.4	globulol	0.1
α-copaene	0.5	viridiflorol	0.3
pinocamphone	0.3	spathulenol	tr
linalool	2.9	γ-eudesmol	0.8
pinocarvone	0.1	T-cadinol	0.3
bornyl acetate	0.9	T-muurolol	0.6
α-fenchol	1.7	δ-cadinol	0.3
terpine-4-nyl acetate	0.3	α-eudesmol	1.3
terpinen-4-ol	0.4	β-eudesmol	1.5

**Table 13:** Compounds identified in the leaf oil of *Backhousia subargentea*.

Compound	%	Compound	%
α-pinene	37.1	borneol	0.4
α-fenchene	0.1	verbenone	0.1
camphene	0.2	piperitone	tr
β-pinene	0.1	carvone	0.1
sabinene	0.03	*trans*-piperitol	0.1
myrcene	tr	δ-cadinene	0.1
α-phellandrene	tr	myrtenol	0.1
α-terpinene	0.04	*trans*-mentha1,(7),8-dien-2-ol	1.1
limonene	24.7	C_10_H_16_O	tr
1,8-cineole	15.7	trans-mentha1,8-dien-6-ol	0.3
isobutyl isobutyrate	tr	p-cymene-8-ol	0.04
γ-terpinene	0.1	C_10_H_16_O	0.1
*E*-β-ocimene	0.2	*cis*-mentha-1,8-dien-6-ol	1.1
p-cymene	0.4	*cis*-mentha-1(7),8-dien-2-ol	1.1
terpinolene	0.2	unknown, mw 148	0.1
isoamyl isovalerate	7.1	C_15_H_24_O	tr
C_10_H_16_O	0.03	palustrol	tr
a butyrate ester	0.02	phenylethyl propionate	0.1
a hexyl butyrate	0..02	C_15_H_26_O	0.02
a hexyl valerate	0..1	C_15_H_26_O	tr
mentha-1,3,8-triene	0.02	C_15_H_26_O	0.1
a hexyl valerate	0.4	C_15_H_26_O	0.04
a hexyl valerate	0.3	E-nerolidol	0.1
α,p-dimethylstyrene	0.03	C_15_H_26_O	0.1
α-campholenic aldehyde	0.1	C_15_H_26_O	0.03
linalool	0.03	globulol	1.0
pinocarvone	0.6	viridiflorol	0.2
fenchol	0.2	C_15_H_26_O	0.1
β-gurjunene	0.1	C_15_H_26_O	0.1
terpinene-4-ol	0.1	spathulenol	0.1
β-caryophyllene	tr	T-cadinol	0.1
aromadendrene	0.7	thymol	tr
a C_10_ acetate	0.1	γ-eudesmol	0.2
alloaromadendrene	0.1	δ-cadinol	0.2
trans-pinocarveol	2.2	carvacrol	0.1
C_10_H_16_O	0.1	α-eudesmol	0.2
C_10_H_16_O	0.1	β-eudesmol	0.3
α-terpineol	1.4	jensenone (17)	<5

**Table 14:** Composition of the leaf oils of the two chemotypes of *Syzygium anisatum*.

Compound	*E*-Anethole Form %	Methyl Chavicol Form %
α-pinene	0.09	0.40
C_10_H_16_	0.06	0.05
1,8-cineole	0.02	0.80
methyl chavicol (18)	4.43	77.4
*Z*-anethole	0.05	0.05
C_15_H_26_O	0.01	0.10
α-farnesene	0.07	0.11
*E*-anethole (19)	95.0	20.0
anisaldehyde	0.01	tr

## Data Availability

Not applicable.

## References

[1] Harrington M.G., Jackes B.R., Barrett M.D., Craven L.A., Barrett R.L. (2012). Phylogenetic revision of Backhousieae (Myrtaceae): Neogene divergence, a revised circumspection of *Backhousia* and two new species. Aust. Syst. Bot..

[2] Hooker W.J., Harvey W.H. (1845). *Backhousia myrtifolia*, Myrtle-leafed *Backhousia*. Curtiss Bot. Mag..

[3] Gildemeister E., Hoffmann F. (1963). Die Ätherischen Öle. Band III C.

[4] Penfold A.R. (1922). The essential oils of *Backhousia myrtifolia* Hooker et Harvey. Part I. J. Proc. Roy. Soc. NSW.

[5] Penfold A.R., McKern H.H.G., Spies M.S. (1953). The essential oils of *Backhousia myrtifolia* Hooker et Harvey. Part II. The occurrence of physiological forms. J. Proc. Roy. Soc. NSW.

[6] Hellyer R.O., McKern H.H.G., Willis J.L. (1955). The essential oil of Backhousia myrtifolia Hooker et Harvey. Part III. Single tree studies of physiological forms from Queens-land. J. Proc. Roy. Soc. NSW.

[7] Jones T.G.H., Lahey F.N. (1938). Essential oils from the Queensland flora. Part XIII. Backhousia hughesii. Proc. Roy. Soc. QLD.

[8] Lahey F.N., Jones T.G.H. (1939). Essential oils of the Queensland flora—Part XV. Backhousia bancroftii and Daphnandra rapandula. Proc. Roy. Soc. QLD.

[9] Penfold A.R. (1923). The essential oil of *Backhousia angustifolia*. Part I. J. & Proc. Roy. Soc. NSW.

[10] Birch A.J. (1951). β-Triketones. Part I. The structures of angustinone, dehydroangustione, calythrone and flavaspidic acid. J. Chem. Soc..

[11] Birch A.J., Elliott P., Penfold A.R. (1954). Studies in biosynthesis. VI. Angustifolionol. Aust. J. Chem..

[12] Birch A.J., Elliott P. (1956). Studies in relation to biosynthesis VIII. Tasmanone, dehydroangustione, and calythrone. Aust. J. Chem..

[13] Cannon J.R., Corbett N.H. (1962). Physiological forms of *Backhousia angustifolia*. F. Muell. Aust. J. Chem..

[14] Brophy J.J., Clarkson J.R., Fookes C.J.R. (1989). Angustifolenone, a ketone from *Backhousia angustifolia*. Phytochemistry.

[15] McKern H.H.G. (1949). A note on the essential oil of *Backhousia anisata* Vickery and the occurrence of anethole. J. Proc. Roy. Soc. NSW.

[16] Brophy J.J., Boland D.J. (1991). The leaf essential oil of two chemotypes of *Backhousia anisata* Vickery. Flav. Fragr. J..

[17] Southwell I.A., Birmingham R.E., Brophy J.J. (1996). Aniseed myrtle-leaf quality. Aust. Rainforest Bushfoods Industry Assoc. Newsletter.

[18] Blewitt M., Southwell I.A. (2000). *Backhousia anisata* Vickery, an alternative source of (E)-anethole. J. Essent. Oil Res..

[19] Craven L.A., Biffin E. (2005). *Anetholea anisata* transferred to, and two new Australian taxa of, *Syzygium* (Myrtaceae). Blumea.

[20] Wilson P.G., O’Brien M.M., Quinn C.J. (2000). Anetholea (Myrtaceae), a new genus of *Backhousia anisata*: A cryptic member of the Acmena alliance. Aust. Syst. Bot..

[21] Blogg J.K. (1920). Some Australian essential oils. Sci. Ind..

[22] Penfold A.R., Morrison F.R., Willis J.L., McKern H.H.G., Spies M.C. (1951). The occurrence of a physiological form of *Backhousia citriodora* F. Muell. and its essential oil. J. Proc. Royal Soc. NSW.

[23] Penfold A.R., Morrison F.R., Willis J.L., McKern H.H.G., Spies M.C. (1950). The occurrence of a physiological form of *Backhousia*
*citriodora* F. Muell. containing Laevo-citronellol. Aust. J. Sci..

[24] Doran J.C., House A.P.N. (1996). Improvement of *Backhousia citriodora*. Aust. Rainforest Bushfood Ind. Assoc. Newsletter.

[25] Doran J.C., Brophy J.J., Lassak E.V., House A.P.N. (2001). *Backhousia citriodora* F. Muell.—Rediscovery and chemical characterisation of the L-citronellal form and aspects of its breeding system. Flavour Fragr. J..

[26] Southwell I.A. (2021). *Backhousia citriodora* F. Muell. (Lemon Myrtle), an unrivalled source of citral. Foods.

[27] Penfold A.R. (1924). The essential oil of *Backhousia sciadophora* (N.O. Myrtaceae) (F.v.M.). J. Proc. Roy. Soc. NSW.

[28] Bean A.R. (2003). *Backhousia oligantha* (Myrtaceae). A new species from Queensland. Austrobaileya.

[29] Brophy J.J., Goldsack R.J., Fookes C.J.R., Forster P.I. (1995). Leaf oils of the genus *Backhousia* (Myrtaceae). J. Essent. Oil Res..

[30] Guymer G.P. (1988). A new species of *Backhousia* Hook. & Harvey (Myrtaceae) from Queensland and a reappraisal of *Backhousia floribunda* A.J. Scott. Austrobaileya.

[31] Brophy J.J., Goldsack R.J., Forster P.I. (1994). The essential oils of *Chorcarpia leptopetala* (F. Muell.) Domin and *C. subargentea* (C.T.White) L.A.S. Johnson (Myrtaceae). Flav. Frag. J..

[32] Babushok V.I., Linstrom P.J., Zenkevich I.G. (2011). Retention Indices for Frequently Reported Compounds Plant Essential of Oils. J. Phys. Chem. Ref. Data.

[33] Henderson R.J.F. (1994). Queensland Vascular Plants: Names and Distributions.

[34] Atkinson N., Brice H.E. (1955). Antibacterial substances produced by flowering plants. 2. The antibacterial action of essential oils from some Australian plant. Aust. J. Exp. Biol..

[35] Potts K.T., Roy S.K. (1965). Triterpenoid constituents of *Backhousia angustifolia* F. Muell. Aust. J. Chem..

[36] Brophy J.J., Craven L.A., Doran J.C. (2013). Melaleucas: Their Botany, Essential Oils and Utilization.

[37] Brophy J.J., Goldsack R.J., Forster P.I., Clarkson J.R., Fookes C.J.R. (1996). Mass spectra of some β-triketones from Australian Myrtaceae. J. Essent. Oil Res..

[38] Francis W.D. (1981). Australian Rain-Forest Trees.

[39] Dastlik K.A., Ghisalberti E.L., Jefferies P.R.F. (1989). Phloroacylphenonesins in the essential oil of *Thryptomene saxicola*. Phytochemistry.

[40] Doimo L. (2001). Iso-citrals and iso-geraniols in lemon-myrtle (*Backhousia citriodora* F. Muell.) essential oils. J. Essent. Oil Res..

[41] Ford A.J., Craven L.A., Brophy J.J. (2005). *Backhousia enata* A.J.Ford, Craven & J.Holmes (Myrtaceae), a *New Species* of Melaleuca (Myrtaceae), from north-eastern Queensland. Austrobaileya.

[42] Brophy J.J., Goldsack R.J., Craven L.A., Ford A.J. (2007). Leaf oil of *Backhousia enata* (Myrtaceae). J. Essent. Oil Res..

[43] Floyd A.G. (1989). Rainforest Trees of Mainland Southeastern Australia.

[44] Forster P.I., Bostock P.D., Bird L.H., Bean A.R. (1991). Vineforest Plant Atlas for South-East Queensland.

[45] Boland D.J., Brophy J.J., Fookes C.J.R. (1992). Jensenone, a new ketone from *Eucalyptus jensenii* Maiden. Phytochemistry.

[46] Leigh J.D., Briggs J.H., Hartley W. (1981). Rare and Threatened Australian Plants: Australian National Parks and Wildlife Service.

[47] Brophy J.J., House A.P.N., Boland D.J., Lassak E.V., Brophy J.J., House A.P.N., Boland D.J. (1991). Digests of the essential oils of 111 species from northern and eastern Australia. Eucalyptus Leaf Oils-Use, Chemistry, Distillation and Marketing.

[48] Adams R.P. (2007). Identification of Essential Oils Components by Gas Chromatography/Quadrupole Mass Spectroscopy.

